# Case Report: Malignant peritoneal mesothelioma with TFG-ROS1 fusion responds to crizotinib

**DOI:** 10.3389/fonc.2025.1617457

**Published:** 2025-10-16

**Authors:** Wei Ye, Peng Li, Cong Fu, Tong Zhou

**Affiliations:** Department of Oncology, Changzhou Cancer Hospital, Changzhou, China

**Keywords:** malignant peritoneal mesothelioma, TFG-ROS1 rearrangement, ROS1 p.K1991N, crizotinib, resistance, case report

## Abstract

**Background:**

Malignant peritoneal mesothelioma (MPM) is an exceptionally rare tumor type, and its molecular properties are poorly understood. In recent years, gene rearrangement has been found in a subset of MPMs. However, *ROS1* rearrangement has not been previously reported in MPM.

**Case presentation:**

Here, we present the first case report of MPM with *TFG-ROS1* rearrangement in a 56-year-old female with no history of asbestos exposure. The patient did not respond to immunotherapy but exhibited sensitivity to crizotinib with a progression-free survival (PFS) of 6 months. Importantly, we identified *ROS1 p.K1991N* as a potential acquired drug resistance mutation to crizotinib, suggesting that entrectinib may serve as a targeted therapy to overcome this resistance mechanism.

**Conclusion:**

*ROS1* rearrangement could potentially represent a novel driver mutation in MPM, especially in female adults. This case report illustrates the benefits of molecular detection in MPM and underscores the potential for lessons learned from other solid tumors to inform treatment strategies for rare diseases.

## Introduction

Malignant peritoneal mesothelioma (MPM), a type of mesothelioma, is a rare and fatal disease. However, its incidence is much lower than that of pleural mesothelioma, accounting for only 7% to 20% of mesothelioma cases (1). MPM may occur at any age, with the median age at diagnosis typically around 50 years, and it is more common in men (2). The clinical symptoms of MPM are highly atypical and may include abdominal pain, bloating, weight loss, ascites, and anorexia. In a minority of patients, MPM may be accompanied by fever and/or intestinal obstruction (3). Chemotherapy is the standard treatment for advanced MPM, yet the prognosis remains poor (4). Although pemetrexel and gemcitabine have shown a longer median overall survival (OS) as an alternative, treatment-related toxicity is significant, limiting their clinical use in MPM patients (5). Therefore, ongoing efforts should focus on developing new treatment regimens to improve the prognosis of MPM patients.

The emergence of targeted therapy and immunotherapy based on molecular characteristics has significantly improved the prognosis of solid tumors, particularly in lung cancer patients. However, due to the low incidence of MPM, few comprehensive genomic studies have been conducted to date. According to published research data, the most commonly mutated genes in MPM are *BAP1* (47.9%), *NF2* (26.5%), *CDKN2A* (25.9%), *CDKN2B* (19.5%), and *PBRM1* (15.8%) (6). Fusion gene variants are extremely rare in MPM. Nevertheless, it is worth noting that ALK gene rearrangement and *EWSR1/FUS-ATF1* fusion have been reported in young MPM patients (7, 8), and there is even speculation that *ALK* fusion-positive mesothelioma tends to predominate in children, women, and abdominal cancers. However, other fusion gene variants have not been reported.

This case report presents, for the first time, the clinical course of a 56-year-old woman diagnosed with Malignant Peritoneal Mesothelioma (MPM) with *TFG-ROS1* fusion. She exhibited a positive response to crizotinib, with a survival time exceeding 17.3 months.

## Case presentation

**Figure 1:**
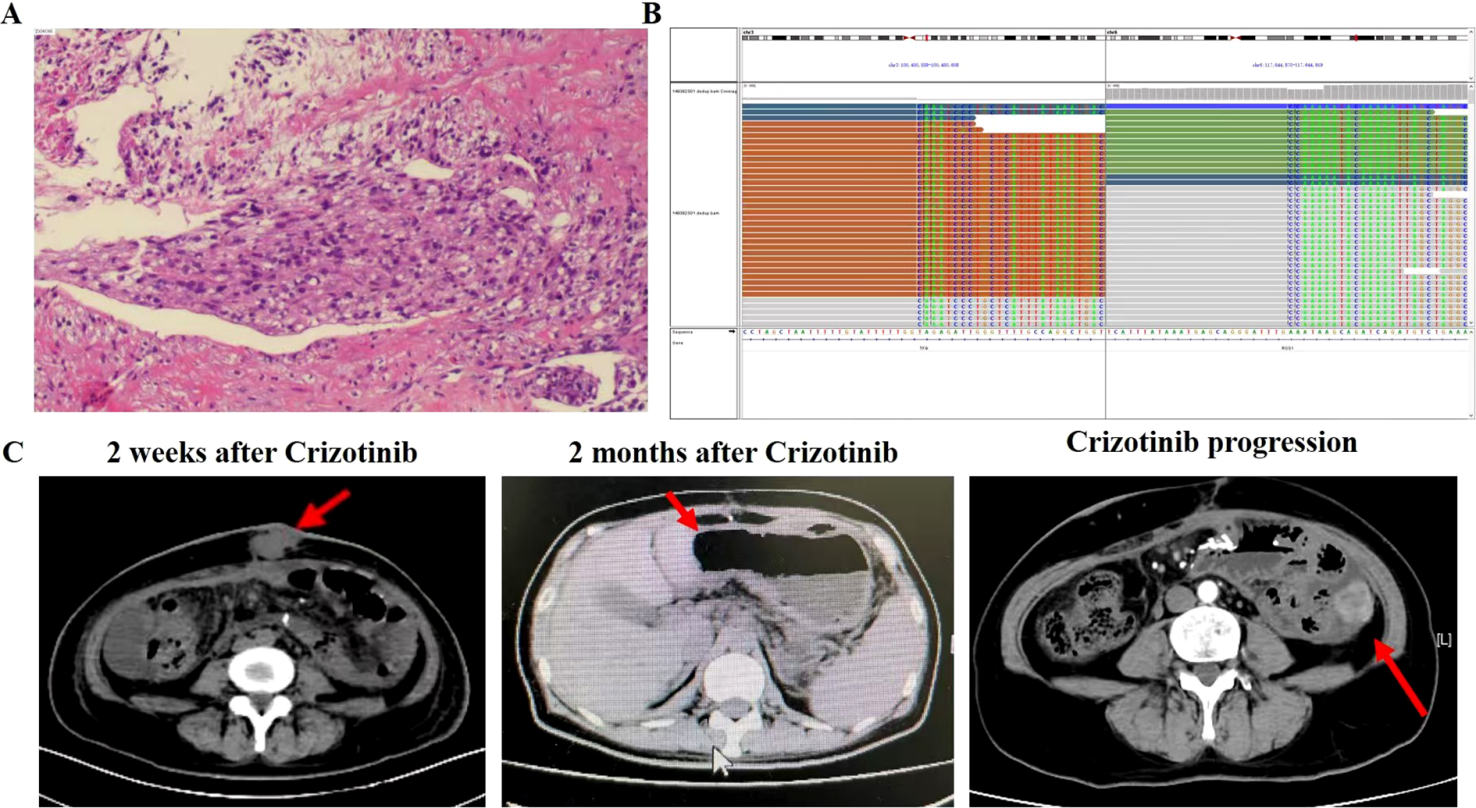
Patient disease diagnosis and treatment of the patient’s disease. **(A)** Pathologic diagnosis result. **(B)** Next-generation sequencing results showing break point of *TFG-ROS1* fusion. **(C)** CT images of lesion changes after crizotinib treatment, as well as new lesions in the left colon as treatment progressed. The red arrow indicates the tumors.

In January 2022, a 56-year-old woman with no history of asbestos exposure presented at a local hospital with complaints of abdominal pain. The patient had a medical history of type 2 diabetes mellitus and underwent cervical cancer surgery 10 years prior. An abdominal CT scan indicated the presence of an abdominal mass with signs of intestinal obstruction. The patient was placed on fasting and provided with nutritional support, followed by an abdominal tumor reduction operation on January 25, 2022. Postoperative pathology revealed MPM (**Figure 1A**). Immunohistochemical analysis showed the tumor to be positive for cytokeratin 5.2, p63, and Ki67, but negative for cytokeratin pan and epithelial membrane antigen. Next-generation sequencing (NGS) confirmed the presence of *TFG-ROS1* fusion (**Figure 1B**), with no other relevant findings. The tumor mutation load was determined to be 2.86 mut/Mb, and PD-L1 was found to be positive (TC>10%). On March 9, 2022, a CT scan revealed a new mass in the anterior abdominal wall and multiple metastases in the liver. Subsequently, the patient underwent one cycle of pembrolizumab combined with pemetrexed chemotherapy on March 13, 2022.After treatment, the patient experienced grade 4 bone marrow suppression, which was managed with symptomatic treatment, and she was subsequently transferred to our hospital for further care.

The physical examination revealed an ECOG PS score of 3, along with anemia, emaciation, listlessness and edema of both lower limbs. A CT imaging showed a mass in the left upper abdomen. Laboratory tests revealed reduced hemoglobin (80 g/L), platelets (36×10^9/L), albumin (24 g/L), and cholinesterase (1500 U/L), with no abnormalities in blood tumor markers. Initially, the patient received active treatment to elevate white blood cell and platelet counts. Following a multidisciplinary team (MDT) case discussion, it was decided to initiate crizotinib (250 mg bid) treatment. After 1 week of treatment, a CT scan revealed a partial response (**Figure 1C**). Subsequently, hemoglobin, platelets, albumin, and cholinesterase levels returned to normal, and the ECOG PS improved to 1. The main adverse event reported was grade 2 gastrointestinal discomfort. Six months after starting crizotinib, progression of the pelvic anastomosis and left paracolon was observed on a CT scan on October 12, 2022 (**Figure 1C**).

In order to explore new therapeutic strategies, NGS detection was conducted on the pelvic mass, which revealed *TFG-ROS1* rearrangement and acquired *ROS1 p.K1991N* mutation. Subsequently, the patient was prescribed oral lorlatinib. Unfortunately, in January 2023, the patient experienced nausea, abdominal distension, and abdominal pain. A follow-up CT scan indicated the presence of multiple soft tissue masses in the abdomen and pelvis, some of which were new lesions, suggesting acquired resistance to lorlatinib. Subsequent treatments with entrectinib and local radiotherapy were administered, but the patient’s condition continued to deteriorate. NGS was performed once more on a puncture sample of the abdominal mass, revealing novel point mutations (*p.L2086F* and *p.S1986F*) of the *ROS1* gene, along with the absence of the *ROS1 p.K1991N* mutation.

**Figure 2:**
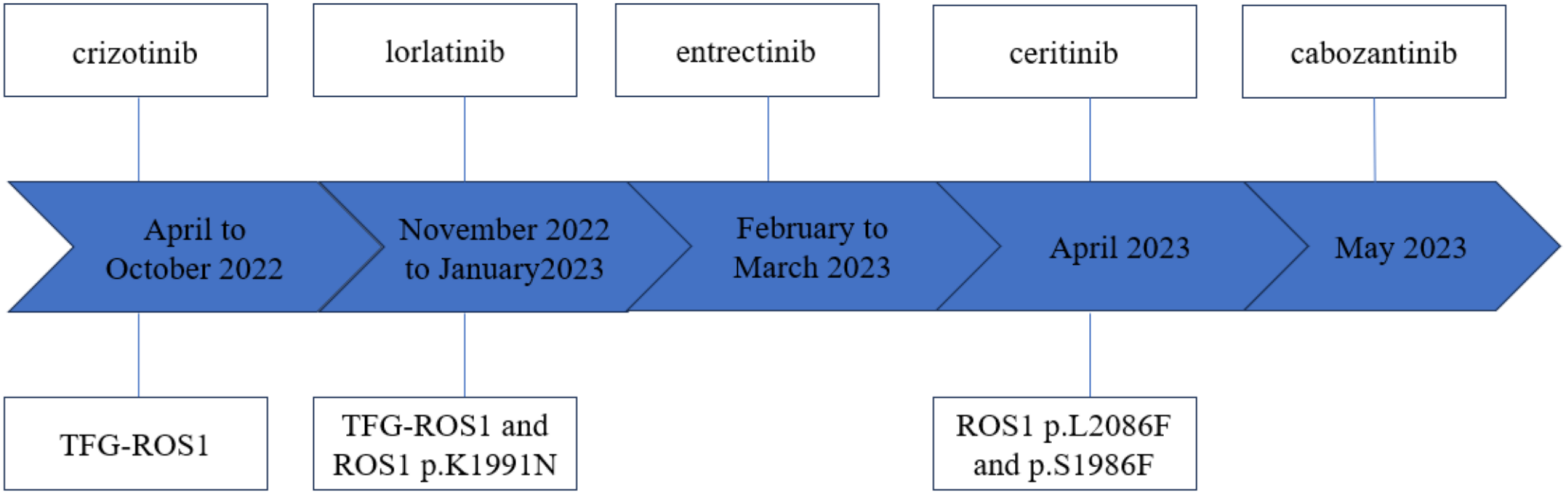
Patient’s treatment timeline and mutation findings.

Given that the patient remained an MPM patient with *ROS1* rearrangement, ceritinib treatment was subsequently attempted. However, a progressive increase in jaundice occurred during the treatment, leading to discontinuation of the drug. Subsequently, the patient received cabozantinib treatment, which resulted in CT imaging showed partial shrinkage of the abdominal mass after one week of treatment. Nevertheless, the drug had to be discontinued due to bleeding and hypertension. The patient was discharged from the hospital in a coma on June 27, 2023. The complete treatment timeline of the patient is shown in **Figure 2**.

## Discussion

To the best of our knowledge, this is the first case report describing the clinical outcomes of *ROS1* rearrangement in MPM. MPM is a rare and aggressive form of mesothelioma with an annual incidence of 7 per million and a poor prognosis, typically resulting in a median survival of 6–12 months (9). Importantly, MPM has garnered increased attention in recent years due to its molecular properties and actionable targets.

ROS1 rearrangement are well-established oncogenic drivers in non-small cell lung cancer (NSCLC), where they confer sensitivity to tyrosine kinase inhibitors (TKIs) such as crizotinib. However, acquired resistance inevitably develops, often through secondary mutations in the ROS1 kinase domain (e.g., ROS1 G2032R), activation of bypass signaling pathways such as EGFR or KIT, or epithelial-to-mesenchymal transition (10).Despite these challenges in NSCLC, the role of ROS1 rearrangements in MPM remains largely unexplored.

This report details the case of a 56-year-old female with MPM who was found to have *TFG-ROS1* fusion through NGS testing. The patient achieved a partial response following treatment with crizotinib. This report presents a potential new treatment option for certain MPM patients with *ROS1* gene rearrangement.

Immune checkpoint inhibitors (ICIs) have been integrated into treatment regimens for patients with diverse solid tumors, with mesothelioma trials demonstrating promising results. However, patients with MPM have typically been excluded from these trials (11, 12). Our patients experienced disease progression after receiving pembrolizumab combined with pemetrexed chemotherapy, showing limited or no efficacy. Currently, there is still no definitive guidance on the role of chemotherapy or immunotherapy in MPM patients with *ROS1* rearrangement.

The proto-oncogene ROS1 encodes a receptor tyrosine kinase implicated in a range of cancers affecting both adult and pediatric patients. ROS1-directed tyrosine kinase inhibitors (TKIs) have demonstrated therapeutic efficacy against these cancers. However, nearly all cases of cancer with associated cancer-causing drivers develop resistance to TKIs following initial treatment. Drug resistance has emerged as a primary factor limiting the clinical utility of TKIs and poses a pressing challenge impacting the survival of patients with advanced tumors. Encouragingly, ongoing research is progressively uncovering the molecular mechanisms underpinning acquired resistance to TKIs. As this knowledge continues to evolve, novel therapeutic strategies are being employed to prolong the lives of patients with advanced tumors.

The patient described in this report was diagnosed with MPM featuring *ROS1* rearrangement. Initially, she responded to crizotinib treatment, but her condition eventually deteriorated. We identified an acquired mutation, *ROS1 p.K1991N*, in her pelvic mass, leading to resistance to crizotinib. This represents the first documented case confirming that the *ROS1 p.K1991N* mutation can induce acquired resistance to crizotinib in a clinical context, and suggests that entrectinib may serve as a targeted drug to overcome this resistance mechanism. Additionally, *ROS1 p.S1986F* and *p.L2086F* have been reported as one of the resistance mechanisms of crizotinib and lorlatinib, which aligns with our findings (13, 14).

Targeted therapy utilizing TKIs that bind to the receptor tyrosine kinase domain of the ROS1 protein has demonstrated effectiveness against cancers harboring these mutations and has been approved for therapeutic use. Considering the potential benefits of TKIs targeting *ROS1*, screening for *ROS1* rearrangement in patients with MPM should be contemplated. Based on all available published data and our study, *ROS1* rearrangement may be more prevalent in female patients (15). We speculate that the estrogen environment within the female body may provide a more favorable microenvironment for the occurrence of ROS1 rearrangement or for the survival of tumor cells driven by ROS1 rearrangement. However, this requires further experimental verification (16).Whether all MPM patients should undergo testing for *ROS1* rearrangement remains an open question that warrants further investigation. Nevertheless, we recommend *ROS1* rearrangement testing for all female patients with MPM due to its therapeutic significance. Naturally, to substantiate the efficacy of ROS1 inhibitors in MPM, a multicenter trial is imperative given the rarity of this disease.

To the best of our knowledge, this is the first instance of consecutive treatment with multiple different ROS1 inhibitors in *TFG-ROS1* rearranged MPM. MPM with *TFG-ROS1* fusion represents a highly uncommon malignant tumor. Our case demonstrates that insights gained from lung cancer treatment can be extrapolated to another disease entity. Currently, there is no established treatment for this rare driver mutation in MPM, and further sample analysis is warranted for validation in the future.

## Data Availability

The original contributions presented in the study are included in the article/supplementary material. Further inquiries can be directed to the corresponding author/s.
